# Global partnerships in combating tropical diseases: assessing the impact of a U.S. withdrawal from the WHO

**DOI:** 10.1186/s41182-025-00722-8

**Published:** 2025-03-10

**Authors:** Ikponmwosa Jude Ogieuhi, Victor Oluwatomiwa Ajekiigbe, Stephen Olaide Aremu, Victory Okpujie, Peace Uchechi Bassey, Adetola Emmanuel Babalola, Pelumi Gbolagade-Jonathan, Chidera Stanley Anthony, Ifeoluwa Sandra Bakare

**Affiliations:** 1 Siberian State Medical University, Tomsk, Russia; 2 Ladoke Akintola University of Technology, Ogbomosho, Nigeria; 3 Global Health and Infectious Disease Control Institute, Nasarawa State University, Keffi, Nasarawa Nigeria; 4 University of Benin, Ugbowo, Nigeria; 5 School of Public Health, University of Port Harcourt, Port Harcourt, Nigeria; 6 Kornberg School of Dentistry, Temple University, Philadelphia, USA; 7 University of Ibadan, Ibadan, Nigeria; 8 University of Calabar, Calabar, Nigeria; 9 Ternopil State Medical University, Ternopil, Ukraine

**Keywords:** Tropical diseases, Africa, Mortality, WHO, Collaborations, Public health

## Abstract

**Background:**

Annually, tropical diseases are a major cause of mortality; for instance, in 2019, neglected tropical diseases (NTDs) caused 150,000 deaths and 19 million DALYs, with sub-Saharan Africa bearing over half the burden and the other concentrations in Asia and South America. Their impact, though significant, is lower than ischemic heart disease and respiratory infections. The World Health Organization is critical in combating these tropical diseases through surveillance, information campaigns and health promotion. Through international collaborations and initiatives, tropical diseases have been relatively mitigated; for example, global initiatives eradicated smallpox (1980), cut polio cases by 99% (1988–2022), and reduced Guinea worm cases from 3.5 million (1986) to 14 (2023), while NTD prevalence dropped significantly from 1990 to 2020.

Main body

The potential departure of a major player like the United States, the largest WHO donor, which contributed $1.284 billion (20% of its budget) in 2022–2023, surpassing the Gates Foundation ($689M), Gavi ($500M), and the EU ($412M), and its potential withdrawal threatens WHO’s financial stability, jeopardizing emergency responses, disease prevention, and global health initiatives, urging stakeholders to reinforce global health systems. Governments, international organizations, and private partners must work together to create strong, flexible frameworks that prioritize prevention, research, and equitable healthcare delivery. By fostering collaboration, transparency, and mutual accountability, the global health community can continue to make progress toward eliminating the burden of major tropical diseases such as malaria and Dengue fever, among others. Failure to do so could reverse hard-won gains such as the 99% reduction in polio cases since 1988, the near-eradication of Guinea worm disease (from 3.5 million cases in 1986 to 14 in 2023), and declining NTD burdens, leading to resurgence and increased mortality among vulnerable populations worldwide, with devastating consequences for millions of people throughout the world.

**Conclusions:**

This review examines the role of countries and organizations in fighting tropical diseases, with a perspective on the potential consequences of the U.S. exit from the WHO. We also discuss the importance of cross-border collaborations in fighting tropical diseases, healthcare systems strengthening efforts, and a call to strengthen efforts through other sources of funding and collaborations.

## Background

The recent United States (U.S.) political change in administration has led to the process of withdrawal of the country from the World Health Organization (WHO) [1]. Formed in the aftermath of World War II (WWII), the WHO has emerged as the leading international health agency responsible for tracking outbreaks of disease around the globe and also provides resources for disease control and continuous research [2]. While it is reported that a significant proportion, about 22% of its financial revenue, comes from the U.S., the country has pulled out amidst financial and geopolitical friction [3].

Tropical diseases account for 15 million deaths annually, accounting for almost 25% of global mortality [4]. These diseases are most prevalent in low- and middle-income countries, particularly in regions with warmer climates, such as sub-Saharan Africa, South Asia, and parts of South America. Some examples, including malaria and cholera, are directly sponsored by climate factors like humidity and temperature, which influence vector survival and pathogen transmission [4, 5].

Meanwhile, others are influenced by poor healthcare infrastructure and poverty as against climate alone. These include polio and HIV, which remain endemic in several regions in the tropics due to low vaccination coverage and challenges with healthcare access [6]. Global organizations, including the WHO, play critical roles in active and passive surveillance and providing public health measures to prevent the global spread of these conditions while re-evaluating awakening against tropical diseases such as dengue, trypanosomiasis, dracunculiasis, onchocerciasis, and so on, at the national or regional level [7]. There have also been strides towards total eradication of some of the diseases (e.g., wild polio in Nigeria) using grassroots vaccination and other holistic primordial/primary strategies [8, 9].

International collaborations have recorded remarkable successes in reducing the risk factors associated with tropical diseases in multiple countries [5, 10–13]. These successes have led to stronger health institutions in many LMICs, bridging the gap between more developed nations and developing regions while setting the foundation for sustainable health systems across highly burdened regions like Africa [7]. Also, the growing interconnectedness of global populations highlights the significance of these efforts, as the fight against tropical diseases not only benefits endemic zones, but also strengthens global health security by reducing disease transmission across borders [10, 13].

In LMICs, donor agencies and charity-led international aid-driven approaches to healthcare frequently encourage reliance rather than independence. Although traditional aid mechanisms offer temporary relief, it undermines national efforts by diverting resources and establishing alternative institutions that harm local health systems [14]. Short-term fixes do not address systemic problems like infrastructural deficiencies and lack of workers. Long-term impact may also be limited since donor-driven priorities could not coincide with local needs. Government accountability and investment in long-term healthcare solutions are discouraged by an over-reliance on outside assistance [15]. Furthermore, donor’s influence often sharpens African health diplomacy since external players dictate the recipient nation’s health priorities in a manner that may not align with local needs [16]. Donor-driven interventions may lead to fragmented healthcare systems that struggle with sustainability once external funding is withdrawn. To create resilient, self-sustaining healthcare systems, efforts should be directed towards empowering local institutions, bolstering health policy, and increasing capacity rather than sustaining dependency.

In this review, we explore the role of international organizations, particularly WHO-led ones, in combating tropical diseases. Additionally, we evaluate the potential ramifications of a diminished U.S. presence in the organization, focusing on funding shortfalls, research stagnation, and weakened health interventions in low and middle-income countries. Finally, we provide future directions, recommendations, and action toward the fight against tropical diseases, even amid shifting geopolitical landscapes.

### International collaborations in tropical disease control

For the past five decades, international Health and control of tropical diseases have been sustained through alliances between global agencies and organizations in public health and biomedicine [17]. The earliest were the foreign missionaries from Europe (particularly the United Kingdom [18], Spain [19], and Portugal [20]) who took health outreaches to their colonies to address the burden of infectious diseases such as cholera, measles, smallpox, and several others [21]. Also, the Rockefeller Foundation led similar early initiatives to assist nations highly burdened with tropical diseases before the advent of programs set up by the colonial authorities to improve the health among indigenous populations and the early efforts of the Pan American Health Organization (PAHO) [22–25].

The United Nations (U.N.) came into existence in 1945 after the end of WWII based on the propositions of international leaders to create a new international organization to maintain peace and ensure the well-being of humans [26]. WHO was founded in 1948 as a major arm of the U.N. to direct and coordinate the authority's efforts on international health [27]. The world has been plagued with several public health challenges, including pandemics, antimicrobial resistance, malnutrition, climate change, non-communicable diseases, and emerging infections since 1948. However, through the aid and resources provided by the WHO through financial support, immunizations, technological know-how, emergency response, and policy recommendations, many of these burdens have been addressed, and significant strides have been made in medical science and population health globally [17, 28]. Notable interventions include the launching of the Expanded Programme on Immunization (EPI) in 1974, which enhanced global vaccine coverage and aided the eradication of smallpox in 1980. The Millennium Development Goals (MDGs) is another initiative that contributed to the decline in maternal and child mortality and combated major tropical diseases, especially malaria and HIV/AIDS, between 2000 and 2015 [29]. To complement these efforts, since 2015, the Sustainable Development Goals (SDGs) have driven the fight against climate-related health threats, disease preparedness, and universal health coverage [30]. Also, deaths attributed to non-communicable diseases and injuries have reduced worldwide, contributing to an increase in global life expectancy from 67 years in 2000 to 73.33 years in 2024. However, progress in low- and middle-income countries is still slow compared to developed nations. WHO’s concerted efforts have been pivotal in achieving these milestones [28, 31].

### Key global initiatives and partnerships

To promote and safeguard global health, the WHO's efforts are complemented by other members of the U.N. family. The General Assembly, the principal policy-making body of the U.N., oversees all issues related to health and through initiatives led by agencies such as the Joint United Nations Programme on HIV/AIDS (UNAIDS), United Nations Population Fund (UNFPA), and United Nations Children's Fund (UNICEF) [28].

Governments, international organizations, civil society, and the private sector have formed several collaborations to ensure significant results in international health beyond the reach of a single organization. In cooperation with WHO, established global health partnerships that have streamlined efforts in the health sector and adequately respond to challenges affecting regions in the tropics include The Global Fund to Fight AIDS, Tuberculosis and Malaria, and GAVI, the Vaccine Alliance [32].

The Global Fund actively champions the battle against AIDS, tuberculosis (TB), and malaria by raising funds every 3 years, investing over 5 billion US dollars annually to tackle these three tropical diseases.

In 2023, the Global Fund supported antiretroviral therapy (ART) for 25 million people across 100+ countries, covering 63% of the global HIV-positive population and 81% of those on ART. With 39.9 million people living with HIV globally and 7.1 million TB patients, the Global Fund invested 227 million in distributing insecticide-treated mosquito nets [33]. These efforts through the Global Fund partnership have significantly improved population health globally, saving more than 65 million lives [34].

Furthermore, GAVI, the Vaccine Alliance, since its establishment in 2000, has improved access to novel and less-used vaccines, particularly for children in underserved countries such as Nigeria, Ethiopia, Uganda, Mozambique, Pakistan, and some others [35, 36]. GAVI's efforts to introduce innovations to immunization programs and expand access to vaccines have culminated in strengthening immunization systems and improving health outcomes, especially among those who reside in regions highly burdened with infectious diseases such as sub-Saharan Africa [35], Asia [37], and so on. To promote efficiency and accelerate outputs, WHO further assists GAVI's initiatives by sponsoring vaccine-centered research and development, ensuring sustainability in vaccine quality, and developing evidence-based policies to guide the use of vaccines across countries. WHO also supports GAVI in other areas, such as maintaining effective cold chain systems, training clinicians and researchers, and analyzing vaccines after introduction [32].

Other global health partnerships hosted by WHO include the Global Polio Eradication Initiative (GPEI), which national governments and four other partner’s lead; Rotary International, the US Centers for Disease Control and Prevention (CDC), the Bill & Melinda Gates Foundation and UNICEF. Their joint objective is to certify the world as polio-free through their polio-targeted initiatives, particularly in low-resource countries [32]. In other words, the U.S. has been a major contributor to the GPEI, providing $114M in 2022. Its withdrawal from WHO creates a funding gap, challenging eradication efforts. Other partners, like the Gates Foundation, invested $1.2 billion in 2022 and $5 billion in 2023 in health-related programs. This commitment is expected to increase until it compensates for the decline in U.S. contributions [38, 39].

Another partnership is UNAIDS, an organization funded by voluntary contributions from UN Member States, private donors, and global programs like PEPFAR and the Global Fund. While domestic funding now covers 60% of HIV response costs, recent U.S. aid cuts threaten progress, highlighting the need for sustained, diversified financial support to end AIDS by 2030 [40].

Training in Tropical Diseases (TDR) is another global collaborative Programme hosted by WHO and jointly sponsored by UNICEF, the United Nations Development Programme (UNDP), and the World Bank to facilitate the training of professionals, strengthen research capacity in disease-endemic nations, and promote efforts to end diseases attributed to poverty [41].

WHO also leveraged alliances with UNICEF and the Food and Agriculture Organization of the United Nations (FAO) to establish systems to monitor, assess, and regulate healthy diets both at the national and global levels [42]. Since 2000, through the Global Outbreak Alert and Response Network (GOARN), WHO has provided support for operational alertness and feedback to public health events such as pandemics and disasters [43, 44]. The departure of a key actor like the U.S. could weaken outbreak preparedness and global response coordination, particularly in overwhelmed health systems [44].

### The role of non-global North health actors

While U.S. funding cuts threaten global health response, this offers non-traditional global health actors such as humanitarian organizations and philanthropic agencies opportunities to increase that contribution to global health, particularly regarding funding [45].

Notably, the Jack Ma Foundation (JMF) and the Alibaba Foundation played a vital role in global health during the pandemic, in 2020, WHO and JMF gifted COVID-19 essential medical aid to 20 Caribbean Community (CARICOM) countries [46]. In the same year, both foundations donated over 1.5 million laboratory diagnostic test kits and more than 100 tons of COVID-19 prevention and control materials to African Union Member States [47], emphasizing their role in complementing initiatives led by governments. Despite these efforts, there remains a doubt about their long-term sustainability in global health funding.

Furthermore, in over 70 countries, agencies like Médecins Sans Frontières (MSF) have contributed to the provision of emergency medical care to individuals affected by conflict, disasters, and epidemics. In 2023, MSF provided consultations to 16.5 million people, treating 3.7 million malaria cases; however, they are not yet esteemed as major funders like the U.S [48]. Similarly, highly burdened areas, particularly in LMICs, have benefitted from the contributions of religious and faith-based organizations (FBOs) [49] like Muslim Aid [50], who may play a more significant role in bridging the gaps that the potential decline in U.S. donations will create.

### Contributions of the United States to these initiatives

The US, a founding member of WHO in 1948, has sustained the status of a global health leader because of its commitment to driving programs that tackle pressing global health issues. These include the President’s Emergency Plan for AIDS Relief (PEPFAR); the President’s Malaria Initiative (PMI); GAVI, the Vaccine Alliance; the Global Fund to Fight AIDS, Tuberculosis and Malaria; and the Global Health Security Agenda (GHSA) [51]. As a leader in global health, the United States combines strategic diplomacy with selflessness. In addition to saving millions of lives, initiatives like PMI and PEPFAR advance American geopolitical objectives [52]. International health assistance promotes goodwill, increases diplomatic clout, and ensures economic and security benefits, especially in strategically important areas. Although there is no denying the benefits of U.S. global health initiatives, they are not solely selfless. The CIA's employment of a phone vaccination program in Pakistan to find Osama bin Laden is a prominent example [53], which increased vaccine hesitancy and fostered mistrust in health initiatives [54]. Similarly, U.S. health support in conflict areas frequently has two functions: disguising its military or political presence as humanitarian help [55]. This dichotomy presents ethical questions regarding the instrumentalization of health programs for political and security objectives. They serve as soft power tools, influencing international relations, securing trade advantages, and bolstering U.S. influence in global governance.

The US National Academy of Medicine (formerly named The Institute of Medicine) was commissioned in 1970 to consistently report the US commitment to global health [56], striving to improve health for all people in all nations by enhancing well-being and eradicating preventable diseases, disabilities, and deaths. In 2016, about 8.6 million mortalities in LMICs were regarded as preventable through timely and effective healthcare interventions [57]. Through collaborations with other countries and intergovernmental organizations, the U.S. has played significant roles in knowledge-sharing to promote good health outcomes in LMICs such as Nigeria, Bangladesh, Afghanistan and Vietnam [58]. As mentioned previously, a plethora of public health and clinical findings that have a positive impact worldwide have been driven by US-sponsored research bodies such as the National Institutes of Health (NIH) and the National Science Foundation in partnership with renowned university researchers across the globe. These discoveries, including the invention of antiretroviral therapies for HIV/AIDS, vitamin A supplementation, artemisinin-based combination therapies for malaria, and oral rehydration salts for diarrhea diseases, have reduced mortality rates globally [59].

For over seven decades, the U.S., through organizations like WHO and CDC, has saved countless lives and protected people in many regions of the world from health threats like smallpox and polio, contributing a major quota to the fight against tropical diseases. Still, there has also been criticism of U.S. aid initiatives. Some contend that rather than being solely motivated by humanitarian considerations. Its global health projects are frequently influenced by geopolitical goals. Others draw attention to problems with sustainability, reliance, and the preference for American strategic goals over locally driven solutions. Although there is no denying the United States' contributions to global health, there is ongoing discussion on the efficacy and long-term effects of its interventions [59, 60]. Also, the role of the US Department of Defense (DoD) in infectious disease surveillance and research has been considerable. The department’s Global Emerging Infections Surveillance and Response System gathers and analyzes epidemiological data to assist in the control of prominent communicable diseases in underserved nations. At the same time, the Military Infectious Diseases Research Program (MIDRP) develops preventive and curative agents against diseases, including vaccines and new drugs that benefit the US military and other low-resource countries. Both the U.S. military and underprivileged countries benefit from the work of the Military Infectious Diseases Research Program (MIDRP) and the Global Emerging Infections Surveillance and Response System (GEIS), which fight diseases including dengue, Ebola, and malaria [MIDRP]. However, the priority for global health may change if U.S. financing is transferred from WHO to these initiatives. While military initiatives concentrate on biodefense and diseases that affect troops, WHO promotes routine immunization, maternity and neonatal health, and improving the health system. A security-driven strategy would put bioterrorism risks (like smallpox and anthrax) ahead of urgent public health issues like tuberculosis or maternity care [61]. This could hinder disease prevention in vulnerable areas and decrease global health equity. Despite the importance of U.S. military research, an over-reliance on it could result in underfunding vital global health needs [59].

Beyond its contribution to infectious disease prevention, the U.S. supports national tobacco prevention and control programs in LMICs. The CDC and WHO developed the Global Youth Tobacco Survey, which monitors tobacco use among youths in 140 nations [59].

Additionally, the U.S. has been critical in delivering humanitarian assistance to communities devastated by conflict, natural disasters, and disease outbreaks, however, a withdrawal from WHO would result in fewer coordinated international responses to emergencies, it might erode U.S. humanitarian assistance. Money can go to security-driven initiatives at the expense of normal vaccinations and maternity care. Weakening WHO's neutrality could hinder emergency responses and hurt vulnerable populations that depend on international health cooperation since it allows access to sensitive places [62]. The U.S. has also been a strong proponent of WHO’s reform and transformation, promoting efficiency to ensure that WHO is better equipped to address global health challenges, this commitment includes leveraging domestic resources and expertise to strengthen capacity-building and build resilient health systems [63]. However, a major factor in the U.S. pullout has been discontent with the performance and structure of WHO [64]. This action begs whether the withdrawal will speed up or impede reform. On the one hand, by lowering financing and influence from a significant global health actor, the U.S. withdrawal could make WHO less capable of reform. However, taking a step back could pressure WHO to reorganize more successfully to restore its reputation and find new funding sources. There are similarities between the U.S. intent to withdraw from the Paris Climate Agreement (2017–2021) [65] and the U.S. exit from UNESCO (2017) [66], both of which hindered international cooperation while forcing organizations to reevaluate their goals. It remains to be seen if WHO will experience significant reform or disintegration.

Globally, the U.S. maintains a strong presence in WHO collaborating centers and is the top donor, notably contributing US $1.284 billion during the 2022–2023 biennium. Leadership from the U.S. has been instrumental in protecting vulnerable populations funding for emergency response, global health, and disease eradication initiatives. The United States has spearheaded the fight against Ebola outbreaks in West Africa, made a substantial contribution to HIV/AIDS treatment and prevention through PEPFAR, and played a crucial role in the eradication of polio [51].

Furthermore, U.S. organizations such as the Department of Defense, CDC, and USAID offer financial, scientific, and logistical assistance to improve mother and child health, immunization programs, and pandemic readiness in low-income nations. Along with providing earthquake relief in Haiti and continuing support for war-torn regions like Syria and Ukraine, the United States has also mobilized aid for communities impacted by natural disasters, disease outbreaks, and violence. These initiatives have improved global health security and saved millions of lives. By supporting WHO’s emergency health efforts, the U.S. drives global health security, preventing and preparing for future threats to deliver rapid response and recovery [63].

Several other U.S. organizations have led initiatives to eliminate diseases, particularly in sub-Saharan Africa (SSA). For example, the guinea worm disease (dracunculiasis) eradication program was initiated by the Carter Center through the support of the Bill & Melinda Gates Foundation and implemented through the alliance between WHO, CDC, UNICEF, and the diseased countries. The provision of clean water, health education, and treatments through this program has led to the reduction of disease prevalence in Africa by a proportion near 100% [59]. Also, PolioPlus, an initiative of Rotary International, has contributed significantly to the fight against polio, particularly in Africa. Incredibly, in 2024, Nigeria received a donation of $14 million from Rotary International to sustain the country’s polio eradication efforts [67]. Several other African countries have received funding to support vaccination campaigns in previous years. With this strength of support, about 100 million children in the continent have received vaccination against polio since July 2020, and on 25th August 2020, Africa was certified free of wild poliovirus after 4 years without a case of wild polio [68, 69]. Hence, the unilateral decision to halt USAID funding reflects a shift towards isolationism, disregarding the interconnected nature of global health. Such abrupt policy changes undermine collaborative efforts and erode trust between nations and organizations committed to combating infectious diseases. Moreover, the financial void left by the U.S. may not be readily filled by other donors, leading to resource shortages and operational challenges. This approach not only compromises the health of vulnerable populations abroad, but also poses a threat to global health security, as uncontrolled outbreaks can lead to cross-border transmission, affecting populations worldwide.

In addition to the efforts against polio, Africa has also benefited from the U.S. President’s Malaria Initiative (PMI). Before the U.S. withdrawal from WHO, PMI supported 24 countries in SSA that account for almost 90% of the global malaria burden, with a prior intention to expand collaborations to include 3 new countries (Burundi, Gambia, and Togo). The efforts of this initiative contributed to the prevention of more than two billion malaria cases since 2000, benefiting more than 700 million people yearly and preserving 11.7 million lives. PMI also funded more than 220 mosquito surveillance sites and 60 drug resistance monitoring sites and as a result, malaria case rates and death rates decreased in Africa by 27% and 46%, respectively [70, 71].

According to a report from USAID, the U.S. has equally invested funds and resources to drive the fight against COVID-19 in SSA. While USAID partners with governments, civil society, and non-governmental organizations to distribute vaccines, train healthcare workers, and disseminate vital public health education to control the disease, the U.S. White House supported WHO’s aim of vaccinating 70% of the world population. As of 30th June 2022, 44 countries in SSA had received more than 168 million doses of COVID-19 vaccines from the U.S. government. Also, USAID enabled case management and effective infection control in many countries in SSA, improved prevention systems, and provided life-saving liquid oxygen (LOX) to individuals with severe COVID-19 infections in 9 African countries. The agency even consistently improved the responses of partner countries to the economic impacts of the pandemic. The efforts culminated in Africa’s strengthened health systems, vaccine access, and economic stability during the pandemic [72].

Although it is theoretically possible to divert monies that were previously allotted to WHO to USAID or other organizations, the present funding freeze on USAID precludes this possibility in the near future. Even if the freeze is removed, it will take a lot of time and money to create new frameworks and partnerships to replace the cooperative processes WHO has previously supported, which could cause delays in important health activities.

The concurrent suspension of USAID financing and exit from WHO is indicative of a larger trend of U.S. isolationism in international health issues. This strategy undermines global trust and collaboration and upends current health initiatives. Due to the suddenness of these policy changes, impacted nations and organizations have little time to adjust, which could have detrimental effects on the public both immediately and over time.

### The importance of cross-border collaborations in combating diseases

Over the past few decades, the global population has experienced significant growth, increasing from approximately 3 billion in 1960 to over 8 billion in 2025. This expansion is accompanied by enhanced human mobility, with international migrants rising from about 77 million in 1960 to nearly 281 million in 2020 [73, 74]. These trends have intensified global interconnectedness, enabling microorganisms to spread rapidly worldwide. While local outbreaks do not instantly become global, as seen with COVID-19, which took about six months to spread worldwide, the risk of rapid international transmission remains a critical public health concern. This interconnectedness emphasizes the necessity for a collective approach to health [75]. Prioritizing global health is an essential strategy for ensuring economic stability, social equity, and sustainable development globally [76].

The rapid spread of pathogens necessitates agile responses from international organizations. However, the effectiveness of these responses depends on transparency and cooperation among nations. The accusation by former U.S. President Donald Trump that China withheld critical information about COVID-19 highlights the challenges faced by the WHO in ensuring timely and coordinated global action [77]. To address such shortcomings, it is imperative to foster stronger partnerships that not only enable rapid and innovative solutions to global health crises, but also reinforce mechanisms for accountability and information-sharing, ensuring a more effective and sustainable global health response. These partnerships, often between low-income countries and resource-rich nations, play a crucial role in combating tropical diseases that heavily burden certain regions. However, while international collaboration has been instrumental in eradicating diseases like wild polio in Africa, India provides a contrasting example of eliminating the same disease [78]. Through strong domestic initiatives, sustained government commitment, and extensive national immunization campaigns, India managed to eliminate polio largely through its own efforts, reducing reliance on international aid. This demonstrates that while global partnerships can be essential, building self-sufficient health systems is equally critical for long-term disease eradication.

### Potential consequences of the United States withdrawal from World Health Organization

For more than 75 years, the U.S. partook in the founding and activities of the WHO. While the establishment of WHO [27] was initiated by several countries, including China and Brazil, the U.S. has been one of the major forces in this organization while playing significant roles in shaping global health initiatives through funding, training, and provision of resources [79, 80]. However, the U.S. consistently utilizes its involvement to drive geopolitical interests and leverages international health diplomacy to strengthen its global influence. Also, changes in foreign policies and recent U.S. protectionism have contributed to the reduction in multilateral engagement, which fuels doubts about the durability of global health initiatives sustained by U.S. support [81]. The withdrawal of the US from WHO threatens to bring about a significant negative impact across various aspects of global health, which are likely to affect LMICs who are most dependent on international aid disproportionately. In 2019, the majority of the 1.4 million deaths and 74 million DALYs occurred in LMICs [82]. In 2023, LMICs accounted for 98% of the 8.2 million new cases and 1.25 million deaths from TB [83]. Likewise, for malaria, LMICs account for 9 in 10 incidence and deaths [84]. Since these regions battle a significant burden of infectious diseases, weak health systems, and scarce resources, they are often kept in a cycle of dependency on external support, especially from US-based agencies. This overdependence has impacted the establishment of self-sustaining healthcare systems in LMICs, leaving most vulnerable to sudden shifts in donor priorities. For example, in 2020, the Trump administration announced the defunding of WHO due to concerns about the organization’s response to coronavirus [64]. However, Biden later reversed this move. The recent decision to restart defunding ushers in political dynamics, highlighting the risks accompanying LMICs’ over-reliance on external aid. This decision affected global efforts in infectious disease control, nutrition, health education and specialized fields such as global neurosurgery [85]. Furthermore, since the U.S. annually contributes over $400 million to WHO to support disease surveillance and response, research and global health activities like vaccination [86], the complete withdrawal of this support is likely to bring about operational challenges in these programs. Also, prompt outbreak response, vaccine development, and initiatives like PEPFAR, which has saved 25 million lives [52], may be threatened by a withdrawal. While this may weaken the diplomatic power of the U.S., competing nations like China may take advantage of the withdrawal to expand their role in controlling global health [87].

### Funding implications

The U.S. withdrawal from WHO could disrupt the flow of funding, which may affect public health initiatives that rely heavily on U.S. contributions. However, a few organizations affiliated with the U.S. may reallocate donations to other global health programs to mitigate the overall impact. The extent of the disruption is uncertain; it will depend on policy decisions and alternative funding approaches.

Research has shown that public health programs such as GPEI and PEPFAR remained operational in some countries after the duration of funding expired or funding reductions but functioned at reduced capacities. For example, the decrease in GPEI funding in Ethiopia by 70% between 2017 and 2020 led to a 43% decline in staff and program operations [88]. Similarly, while PEPFAR in South Africa is expected to thrive after funding cuts, it is projected that a 50% decrease in funding will mediate 315,000 HIV-related deaths and a decline in the average life expectancy of individuals living with HIV by 2.02 years [89]. This reduced capacity reflected fewer activities conducted for a few individuals or the same population [90]. Furthermore, this loss of financial support often leads to reduced staffing and even the elimination of critical services such as disease surveillance and vaccinations [90]. Local health departments (LHDs) of LMICs, for example, have faced declining budgets over the years, with 81% stagnant funding [91]. This funding gap has forced a shift in program priorities, limiting the ability to implement planned initiatives [90, 91]. The consequences of these gaps are severe to the extent of reported infrastructural loss, weakened technical capabilities, and diminished capacity to maintain public health programs such as STI control (e.g., PEPFAR) and tropical disease initiatives (e.g., the Global Fund, GPEI, and USAID) [90].

Historically, funding withdrawals or shifts by major donor nations have significantly impacted WHO-led global health programs. For example, the UK’s reduction in Official Development Assistance (ODA) for global health between 2020 and 2021 from 0.7% to 0.5% of gross national income (GNI) disrupted WHO-led malaria and neglected tropical disease (NTD) eradication efforts, polio vaccination campaigns, and reproductive health programs [92, 93]. This compelled WHO and its partners to scale down interventions in low-income nations [94].

Similarly, Brazil’s funding reduction for the Pan American Health Organization (PAHO), a WHO regional office, between 2014 and 2016 due to economic crises led to setbacks in WHO-supported immunization and disease control programs. This contributed to yellow fever outbreaks, a resurgence of congenital syphilis, and rising malaria and dengue cases, particularly in 2015 and 2016. These gaps required urgent intervention from alternative donors and multilateral organizations [95].

These examples of how funding shortfalls have forced donor nations and international organizations to prioritize high-burden regions and restructure resource allocation further reinforce concerns about the effects of U.S. withdrawal from the WHO.

### Research and development setbacks

The U.S. withdrawal from the WHO is expected to have some financial impact on research and development in global health. This will remarkably impact clinical trials and drug development due to reduced funding and restricted access to a diverse pool of patients. The WHO, a key global coordinator, facilitates critical projects targeting diseases such as HIV/AIDS, tuberculosis, malaria, and polio, which depend on international collaboration and resource sharing [96–98]. This withdrawal may lead to disrupted progress in combating these health challenges and delaying the development of life-saving treatments because the US is a significant contributor in terms of financial and expert human resources.

Partnerships and collaborations are critical to ensuring equitable access to healthcare, especially in resource limited settings such as countries in sub-Saharan Africa. Although, apart from the US, other developed nations play important roles within the WHO as regards supporting research and development on tropical diseases, the impact of the US cannot be understated. WHO-backed open-access journals might face financial instability and US-based researchers working on infectious tropical diseases could also face barriers in participating in WHO-sponsored open-access collaborations, limiting knowledge transfer and exchange [99, 100].

### Operational challenges in epidemic/pandemic disease surveillance and response

The rollout of COVID-19 vaccines and robust vaccination campaigns improved the vaccination rates in Africa during the pandemic, resulting in a gradual decline in the number of cases. However, operation challenges including the lack of comprehensive models to understand disease dynamics, limited vaccine availability, and distribution barriers hindered effective disease surveillance and response efforts in many developing countries. Notably, the extended SEIRDV (susceptible, exposed, infected, recovered, dead and vaccinated) model was employed in Tunisia and South Africa to estimate the dynamics of infected population and their outcomes, and track the effectiveness of vaccination campaigns, but the adoption of this model in other African countries is still lacking [101].

### Impact on disease-specific programs

Several other far-reaching consequences of the U.S. pullback from WHO include disease-specific programs targeting tropical diseases such as malaria, HIV, and tuberculosis, vaccination programs and NTDs [102]. WHO, in collaboration with other organizations in the US, combats these diseases through funding, technical support, program implementation, and policies to enhance global access to eradicating these diseases, especially in low-income countries [103–105].

The absence of sustainable external support in the face of global health emergencies like the COVID-19 pandemic threatens the control of endemic diseases, particularly worsening the malaria burden of Africa. Since the diversion of the often-limited healthcare resources to the control of outbreaks hinders malaria prevention programs, the years of progress in malaria control will be reversed, potentially exacerbating malaria cases and mortalities [106]. Similarly, programs focused on addressing NTDs like Chagas disease also suffer setbacks when there is a disruption to the flow of NTD-targeted funding [107]. To alleviate these effects, low-income countries must adopt integrated health responses to strike a balance between the control of the pandemic and ongoing programs targeted at tropical diseases.

### Health systems strengthening efforts

Strengthening healthcare systems in endemic regions relies heavily on capacity-building and training programs facilitated by the WHO [108–111]. These programs equip healthcare workers with the skills to manage and prevent outbreaks, improve delivery systems, and build resilient health infrastructures [112]. Beyond training, the WHO plays a crucial role in formulating treatment protocols, coordinating technical assistance, and ensuring that essential medical supplies are distributed equitably [113]. However, reduced contributions from key stakeholders will disrupt these efforts, weakening not only the health workforce but also the broader healthcare infrastructure. These disruptions in health system strengthening efforts will leave many regions vulnerable to preventable diseases, slowing down the progress of global health resilience towards equitable healthcare. Furthermore, the withdrawal of the U.S. from WHO may widen health disparities in regions with weak healthcare systems, compromising global efforts to establish reliable pandemic preparedness and response mechanisms.

### Potential strategies to mitigate the impact

When examining the proposed mitigation strategies for addressing the impact of a potential U.S. exit from the WHO, particularly regarding alternative funding sources and regional collaborations, several critical considerations emerge that warrant careful analysis. Additional support in training, research, and healthcare delivery should be sought through strengthening partnerships with NGOs, academic institutions, and the private sector. There is a need to ensure long-term global health security and equitable healthcare access in vulnerable regions by sustaining investments in health system resilience.

### Role of alternative funding sources

Given the decades of high U.S. contributions to the WHO, the exit of the U.S. from this organization leaves a large funding gap. To offset this, other funding sources need to fill in the gaps. Philanthropic organizations or private individuals like the Bill and Melinda Gates Foundation have already shown they can mobilize resources toward global health challenges [114]. Additionally, these organizations could be crucial in funding ideas and interventions to tackle tropical diseases and research. On the other hand, the heavy reliance on philanthropic funding poses issues of accountability and priority-setting, as private donors’ tiers diseases and regions according to their interests and, as such, could inadvertently ignore other urgent needs [115, 116]. It is important to stress that political decisions may also influence private individuals, and there may be a need for a vast base of donors.

Moreover, other governments, especially in high-income countries, could contribute more to compensate for the gap. For example, the European Union has traditionally supported multilateral health efforts, such as through the European and Developing Countries Clinical Trials Partnership (EDCTP), however, the extent to which this support can compensate for the U.S. withdrawal is uncertain. Analyzing the past contributions of the EU and assessing the possibility of increased funding based on current economic and political trends are areas for future studies to explore [117, 118].

A diversified funding approach, instigated by resources coming from various stakeholders, could reduce dependency on any single source and provide a more balanced priority for global health goals.

Inasmuch as overdependence on external organizations for funding has limited public health interventions, training of healthcare staffs, and vaccine availability in low-resource countries, to establish effective disease surveillance systems and improve control of tropical diseases, we advocate for alternative funding sources to enhance preventive measures, vaccine development and distribution infrastructure. These efforts will mitigate the consequences of the U.S.'s sudden exit from WHO, ultimately bolstering public health responses in many low-resource settings, particularly in Africa [101].

### Strengthening regional collaborations and local capacities

The withdrawal of the U.S. from the WHO also highlights the need for expanding regional cooperation and strengthening local capacities to control tropical diseases. Regional organizations [e.g., African Union (AU) or Association of Southeast Asian Nations (ASEAN)] can play their part in coordinating efforts within their respective regions [119, 120]. Thus, by combining the resources of all the regional actors, the entities will share their best experiences and facilitate interventions across borders to handle outbreaks on the regional levels more efficiently. The withdrawal of the US may also stir governments and stakeholders in SSA to be more accountable as regards their region’s healthcare. It is also time for regions to look beyond a constant dependence on aids. With a strong will and collaborative mindset, tropical regions may develop innovative ways to solve healthcare challenges regarding tropical diseases.

Building local capacity is equally critical. Investments in healthcare infrastructure, workforce training, and community education can empower countries to take ownership of their disease prevention and treatment programs [121]. Furthermore, strengthening local research institutions can drive innovation, ensuring solutions are tailored to specific regional needs.

Even though the existing alliances between universities and biotech firms like the African Vaccine Manufacturing Initiative have shown potential for inventing diagnostic tools and treatments that are affordable [Partnership PAVM], there is a need for more research to assess their scalability and effectiveness in addressing tropical diseases. Besides, developing Public–Private Partnerships (PPPs) at the regional level may attract additional funding and technical expertise [122]. For example, pharmaceutical companies can be encouraged to develop and distribute affordable medications through subsidies [123].

For countries in the tropics, revitalizing the healthcare system after debilitating events such as epidemics, disasters, and national conflicts can be difficult due to weak health insurance schemes, paucity of healthcare professionals and often-limited financial reserves, necessitating the need to reinforce partnerships with international organizations [124].

### Advocacy and diplomatic efforts to encourage re-engagement

To respond to the implications of the exit of the US from the WHO, advocacy and diplomacy are needed. Diplomatic channels can be leveraged to highlight the global interdependence in combating tropical diseases and the necessity of US involvement in this endeavor [5]. By emphasizing how tropical diseases threaten global health security, advocates can present re-engagement not as an act of philanthropy but as a strategic necessity for protecting U.S. interests, including biosecurity and trade stability. Furthermore, multilateral forums—such as G7 and G20 have been used as avenues for global health negotiations, establishment of global health policies, and advocacy for reforms within the WHO, such as during the COVID-19 pandemic. Whether they can successfully mediate U.S. re-engagement in WHO remains an area for further exploration [125, 126].

Additionally, multilateral diplomatic initiatives must focus on multiple targets simultaneously, ranging from congressional leadership to executive branch policymakers, international health experts, and influential non-governmental organizations. These efforts would require using hard empirical evidence about the specific value of cooperation with WHO, such as cost-effective disease prevention, global surveillance capabilities, and strategic preparedness against pandemics [127]. Equally, academia, medical professional associations, and research networks can all contribute to the production and dissemination of research on the economic and humanitarian impact that reduced U.S. engagement would have.

### Leveraging technology and innovation to bridge gaps

Technology and innovation offer transformative solutions to bridge the gaps created by reduced funding and support for tropical disease initiatives. For instance, some of the key transformative approaches include digital health platforms, artificial intelligence-driven epidemiological modeling, and decentralized health information networks in the maintenance of global tropical disease surveillance and intervention capabilities [128]. Currently, sophisticated machine learning algorithms can predict with unprecedented accuracy the pattern of the spread of diseases, allowing for fast and targeted responses across traditional institutional barriers [129]. For example, in Malawi, the introduction of AI-enabled fetal monitoring systems led to an 82% reduction in stillbirths and neonatal deaths. Acquisition and maintenance of such equipment in LMICs often involve partnerships with non-governmental organizations and international donors. Also, Médecins Sans Frontières' introduction of AI-driven snake identification apps in South Sudan illustrates how external aid can strengthen local healthcare capacities [130, 131]. These technological approaches can sustain critical health intelligence infrastructure even in diminished formal institutional collaboration.

Health record systems based on blockchain technologies, such as those piloted in Estonia, have been convincing in improving data security and transparency [132–134]. By creating decentralized, tamper-resistant epidemiological databases, these technologies can facilitate collaborative research and intervention strategies that are less dependent on formal intergovernmental agreements. Moreover, telemedicine platforms and mobile health technologies can democratize access to specialized tropical disease knowledge by extending medical expertise and training into resource-constrained settings [135]. On the other hand, developing a blockchain-based Electronic Health Record (EHR) system costs between $400,000 and $1,500,000, making their adoption challenging in many LMICs, suggesting a need for international collaborations [136, 137]. Future research is required to assess the feasibility and cost-effectiveness of EHR in low-resource settings. Also, cost-reduction strategies, such as open-source blockchain frameworks or PPPs, should be further explored.

In the face of the rising global burden of antibiotic resistance, disproportionately affecting LMICs, innovations like immunotherapy can be leveraged to combat infectious diseases by integrating host-specific therapies. Even though vaccination has been pivotal in controlling diseases such as polio and measles, there is a need to further incorporate innovation into vaccine production. For diseases such as HIV and tuberculosis, against which vaccine strategies have barely yielded remarkable results across at-risk populations, nanotechnology is a promising option to enhance immune system functionality [124, 138].

### Policy and governance considerations

#### The role of national governments in sustaining disease control efforts

In the fight against illnesses, especially tropical ones, the role of national governments cannot be overemphasized [139]. National governments can sustain disease control efforts despite the withdrawal of significant international aid. Cuba is a valid example. After the US trade embargo and the loss of Soviet financial support, Cuba established a strong healthcare system focused on medical education and international medical missions. This did not only generate revenue, but also bolstered its domestic health infrastructure [140]. To combat endemic diseases, they allocate resources for healthcare delivery, play a crucial role in implementing health policies, and promote partnerships with international organizations. Disease control initiatives may be hampered by the lack of strong national governance structures, particularly in low-income nations with little resources [139, 141, 142]. To preserve success, governments must guarantee ongoing investments in disease surveillance systems, staff development, and public health infrastructure. Furthermore, to mobilize resources, provide technical assistance and exchange expertise, strategic alliances with regional and global organizations such as the WHO are still crucial [143–146].

Policies supporting integrating technology and innovative approaches into infectious disease control have shown promise in clinical settings. Since approaches like immunotherapy present potential toxicities, including hematological, gastrointestinal, dermatological, and neurological effects, there remains a need for robust evidence-based policy frameworks that duly address these risks [124]. Efforts can also be made to train healthcare staff on utilizing novel technologies such as AI for disease prediction and outbreak preparedness [128, 129].

#### Potential realignment of global health governance without US participation

The upset to the balance of global health governance by the US’s departure from the organization could result in power vacuums [147, 148]. With emerging economies and regional powers filling the void, this change may spur a realignment of funding sources and leadership. For instance, nations like China, Russia, Canada, Germany, and the U.K. may grow their influence in determining global health objectives, perhaps leading to a more multipolar governance system [2, 3, 80]; however, this is uncertain. Analyzing their current contributions and policy approaches could offer insights into whether a shift towards a multipolar governance system is realistic. This uncertainty is due to their minimum contributions (Germany: $324 million; United Kingdom: $215 million; Canada: $141 million; Russia/China: not listed as a top contributor) compared to the US [149]. Foundations such as the Jack Ma Foundation and Alibaba Foundation have actively participated in global health efforts. For instance, in 2020, they provided essential medical supplies to countries during the COVID-19 pandemic, demonstrating a commitment to addressing health crises. While these contributions are impactful, the scale is limited and sustained impact often requires collaboration with governmental bodies and larger institutions [150]. Also, India's capacity for large-scale vaccine production and distribution positions it as a key player. It is home to a massive biotech industry, the Serum Institute of India, the world's largest vaccine producer by number of doses [151]. However, if global health stakeholders cannot agree on shared objectives, such realignments could lead to disjointed approaches to the fight against tropical diseases. Existing international organizations must modify their structures to foster inclusive decision-making processes that reflect diverse regional needs and perspectives, make room for new participants, and promote inclusive decision-making procedures to reduce these dangers.

#### Opportunities for WHO reform to attract diverse funding and collaboration

The WHO may have to update its operating plans and funding model due to the U.S. exit [2, 5, 80]. The organization's financial stability is vulnerable due to its over-reliance on a small number of significant contributors, including the United States ($1.284 billion, 15% of its budget), the Bill & Melinda Gates Foundation ($689 million, 9.4%), Germany ($600 million, 9%), GAVI Alliance ($500 million, 6%), and the European Commission ($412 million, 5%) [149]. The WHO's resilience can be increased by involving middle-income nations, business sector players, and charitable organizations in diversifying financing sources [152]. Further cooperation in research, capacity-building, and disease prevention programs can also be facilitated by cultivating creative collaborations with academic institutions, non-governmental organizations, and regional alliances [5, 152, 153]. Increased accountability, efficiency, and transparency are organizational reforms that can boost the WHO's reputation and get more support. If the WHO successfully diversifies its funding sources and improves transparency, it could reinforce its capacity to fight tropical diseases. However, further scrutiny is warranted to determine the effectiveness of proposed reforms in achieving these goals.

## Conclusion and future directions

This study highlights the importance of international cooperation in fighting tropical diseases, which mostly impact low-income areas. The potential consequences of the US withdrawing from the WHO expose weaknesses in global health governance and the dangers of disrupting funding, expertise, and international cooperation. As global health challenges evolve, countries must respond by strengthening multinational collaborations and developing robust healthcare systems.

To address the challenges of changing political situations, national governments need to take stronger steps in controlling diseases and ensure that lack of funding does not slow down progress. Working with regional groups like the African Union and ASEAN can help create better ways to fight tropical diseases over time. Additionally, charitable organizations, businesses, and non-profit groups should take on a bigger role in gathering resources and expertise. The WHO should take the opportunity to improve its financial and governance systems to be more transparent and fairer. This will allow better resource mobilization and ensure that the voices of those affected by tropical diseases are included in decision-making.

In the end, no single country can tackle tropical disease control independently. It is important for nations to work together on global health, share new ideas, and fairly distribute resources to make a lasting difference. We should learn from past partnerships to create better strategies for a strong and cooperative global response to these urgent health challenges.

## Data Availability

Data sharing is not applicable to this article as no datasets were generated or analyzed during the current study. No datasets were generated or analyzed during the current study.

## Abbreviations

WHO: World Health Organization
WWII: World War II
PAHO: Pan American Health Organization
UN: United Nations
GAVI: Global Alliance for Vaccines and Immunization (now Gavi, the Vaccine Alliance)
UNAIDS: Joint United Nations Programme on HIV/AIDS
UNICEF: United Nations International Children's Emergency Fund (now United Nations Children’s Fund)
GPEI: Global Polio Eradication Initiative
TDR: Special Programme for Research and Training in Tropical Diseases
CDC: Centers for Disease Control and Prevention
FAO: Food and Agriculture Organization
GOARN: Global Outbreak Alert and Response Network
PEPFAR: President’s Emergency Plan for AIDS Relief
GHSA: Global Health Security Agenda
MIDRP: Military Infectious Diseases Research Program
ASEAN: Association of Southeast Asian Nations
AU: African Union
PPPs: Public–Private Partnerships

## References

[1] Withdrawing The United States From The World Health Organization–The White House. https://www.whitehouse.gov/presidential-actions/2025/01/withdrawing-the-united-states-from-the-worldhealth-organization/. Accessed 25 Jan 2025

[2] Hibbert CM. What the US exit from the WHO means for global health and pandemic preparedness. Northeastern Global News. https://news.northeastern.edu/2025/01/23/us-withdrawn-from-who/. Accessed 25 Jan 2025

[3] Bertozzi S, Lu M. U.S. withdrawal from WHO could bring tragedy at home and abroad | UC Berkeley Public Health. https://publichealth.berkeley.edu/news-media/opinion/withdrawal-from-who-could-bring-tragedy. Accessed 25 Jan 2025

[4] Zumla A, Ustianowski A. Tropical diseases: definition, geographic distribution, transmission, and classification. Infect Dis Clin North Am. 2012;26:195–205. 22632634 10.1016/j.idc.2012.02.007 PMC7135174

[5] Lv S, Zhou XN. Global outreach and networking promotion to accelerate tropical diseases elimination. Infect Dis Poverty. 2024;13:47. 10.1186/s40249-024-01215-2. 38879557 10.1186/s40249-024-01215-2 PMC11180385

[6] Sinumvayo JP, Munezero PC, Tope AT, Adeyemo RO, Bale MI, Mutsaka-Makuvaza MJ, Daba TM, Nyandwi JB, Nzungize L, Mutumwinka D, Omotayo MO, Bello MB, Adedeji KA, Mutesa L, Adedeji AA. Vaccination and vaccine-preventable diseases in Africa, Scientific African, 2024: 24; e02199, ISSN 2468–2276, 10.1016/j.sciaf.2024.e02199.

[7] WHO. Control of Neglected Tropical Diseases. https://www.who.int/teams/control-of-neglected-tropical-diseases/overview. Accessed 25 Jan 2025

[8] Nasiru S-G, Aliyu GG, Gasasira A, Aliyu MH, Zubair M, Mandawari SU, Waziri H, Nasidi A, El-Kamary SS. Breaking community barriers to polio vaccination in Northern Nigeria: the impact of a grassroots mobilization campaign (Majigi). Pathog Glob Health. 2012;106:166–71. 23265374 10.1179/2047773212Y.0000000018 PMC4001576

[9] Nasir UN, Bandyopadhyay AS, Montagnani F, Akite JE, Mungu EB, Uche IV, Ismaila AM. Polio elimination in Nigeria: A review. Hum Vaccin Immunother. 2016;12:658–63. 26383769 10.1080/21645515.2015.1088617 PMC4964709

[10] Hameed M, Najafi M, Cheeti S, Sheokand A, Mago A, Desai S. Factors influencing international collaboration on the prevention of COVID-19. Public Health. 2022;212:95–101. 36272205 10.1016/j.puhe.2022.08.017 PMC9458694

[11] Xu J, Yu Q, Tchuenté L-AT, et al. Enhancing collaboration between China and African countries for schistosomiasis control. Lancet Infect Dis. 2016;16:376–83. 26851829 10.1016/S1473-3099(15)00360-6

[12] Allen I, Lockwood DN, Hay RJ. The Centenary of the Leprosy Relief Association (Lepra) a moment for celebration and reflection†. Trans R Soc Trop Med Hyg. 2024;118:299–303. 38269435 10.1093/trstmh/trad096 PMC11062199

[13] AlKhaldi M, James N, Chattu VK, Ahmed S, Meghari H, Kaiser K, IJsselmuiden K, Kaiser M. Rethinking and strengthening the Global Health Diplomacy through triangulated nexus between policymakers, scientists and the community in light of COVID-19 global crisis. Glob Heal Res policy. 2021;6:12. 10.1186/s41256-021-00195-2 PMC8041471 33845923

[14] OECD. Development co-operation report 2023: debating the aid system. OECD Publish Paris. 2023. 10.1787/f6edc3c2-en.

[15] Kabonga I. Dependency theory and donor aid a critical analysis. Afr J Develop Stud. 2017;46(2):29–39. 10.25159/0304-615X/1096.

[16] Anderson E-L. African health diplomacy: obscuring power and leveraging dependency through shadow diplomacy. Int Relat. 2018;32(2):194–217. 10.1177/0047117817751595.

[17] Meslin EM, Garba I. International Collaboration for Global Public Health. 2016 Apr 13. In: H. Barrett D, W. Ortmann L, Dawson A, et al., editors. Public Health Ethics: Cases Spanning the Globe. Cham (CH): Springer; 2016. Chapter 8. 10.1007/978-3-319-23847-0_8. 28590697

[18] Johnson R. Colonial mission and imperial tropical medicine: Livingstone College, London, 1893–1914. Social History of Medicine. 2010;23(3):549–566. Epub 2010 Jul 27. 10.1093/shm/hkq044

[19] Franco-Paredes C, Lammoglia L, Santos-Preciado JI. The Spanish royal philanthropic expedition to bring smallpox vaccination to the new world and Asia in the 19th century. Clin Infect Dis. 2005;41(9):1285–9. 10.1086/496930. 16206103 10.1086/496930

[20] Amara I. The emergence of tropical medicine in Portugal: the school of tropical medicine and the colonial hospital of Lisbon (1902–1935). Dynamis. 2008;28:301–28 (**PMID: 19230343**). 19230343

[21] Browne SG. The contribution of medical missionaries to tropical medicine. Service-training-research. Trans R Soc Trop Med Hyg. 1979;73(4):357–60. 10.1016/0035-9203(79)90155-x. (**PMID: 400201**). 400201 10.1016/0035-9203(79)90155-x

[22] Fosdick RB. The story of the Rockefeller Foundation. New Brunswick: Transaction Books. 1989.

[23] Good CM. Pioneer medical missions in colonial Africa. Soc Sci Med. 1991;32(1):1–10. 2008614 10.1016/0277-9536(91)90120-2

[24] Marks S. What is Colonial about Colonial Medicine? And what has happened to imperialism and health? Soc Hist Med. 1997;10(2):205–19. 11619493 10.1093/shm/10.2.205

[25] Cueto M. The value of health: a history of the Pan American Health Organization. Washington, DC: Pan American Health Organization; 2007.

[26] Weiss TG. The United Nations: before, during and after 1945. Int Aff. 2015;91(6):1221–35. 10.1111/1468-2346.12450.

[27] Kirigia D, Asante A. United Nations and Global Health. In: Kickbusch, I., Ganten, D., Moeti, M. (eds) Handbook of Global Health. Springer, Cham. 2021. 10.1007/978-3-030-45009-0_119

[28] Global Issues: Health. United Nations. https://www.un.org/en/global-issues/health. Accessed 27 Jan 2025

[29] W.H.O. Millennium Development Goals (MDGs). 2018. https://www.who.int/news-room/fact-sheets/detail/millennium-development-goals-(mdgs). Accessed 21 Feb 2025

[30] U.N.D.P. The SDGs in Action. What are the sustainable development goals?. 2018. https://www.undp.org/sustainable-development-goals. Accessed 21 Feb 2025

[31] World Life Expectancy 1950–2025. Macrotrends. https://www.macrotrends.net/global-metrics/countries/wld/world/life-expectancy. Accessed 27 Jan 2025

[32] Global health partnerships. World Health Organization. https://www.who.int/europe/about-us/partnerships/global-health-partnerships. Accessed 27 Jan 2025

[33] Becker, V. The Global Fund. Results Report 2024. https://www.theglobalfund.org/en/results/. Accessed 21 Feb 2025

[34] The Global Fund to Fight AIDS, Tuberculosis and Malaria. The Global Fund. https://www.theglobalfund.org/. Accessed 27 Jan 2025

[35] GAVI. Africa 2024. https://www.gavi.org/programmes-impact/country-hub/africa. Accessed 21 Feb 2025

[36] GAVI. Eastern Mediterranean 2024. https://www.gavi.org/programmes-impact/country-hub/eastern-mediterranean. Accessed 21 Feb 2025

[37] GAVI. South-East Asia 2024. https://www.gavi.org/programmes-impact/country-hub/south-east-asia. Accessed 21 Feb 2025

[38] Our Partners – GPEI. Polio | Global Eradication Initiative. https://polioeradication.org/who-we-are/partners/. Accessed 27 Jan 2025

[39] Si, A.K. Top Gates Foundation health grantees in 2023. devex. Gates Foundation. 2024. https://www.devex.com/news/top-gates-foundation-health-grantees-in-2023-107128. Accessed 21 Feb 2025

[40] The global partnership for action to eliminate all forms of HIV-related stigma and discrimination. UNAIDS.. https://www.unaids.org/en/topic/global-partnership-discrimination. Accessed 27 Feb 2025

[41] Ogundahunsi OA, Vahedi M, Kamau EM, Aslanyan G, Terry RF, Zicker F, Launois P. Strengthening research capacity–TDR’s evolving experience in low- and middle-income countries. PLoS Negl Trop Dis. 2015;9(1): e3380. 10.1371/journal.pntd.0003380. (**PMID:25569232;PMCID:PMC4287519**). 25569232 10.1371/journal.pntd.0003380 PMC4287519

[42] Partnering with UNICEF | FAO and UN System Partnerships. FAO. https://www.fao.org/partnerships/fao-un-system/UN-Partners/fao-and-unicef/en. Accessed 27 Feb 2025

[43] Salmon S, Brinkwirth S, Loi G, Basseal JM. Global outbreak alert and response network deployments during the COVID-19 pandemic, WHO Western Pacific Region. Western Pac Surveill Response J. 2024;15(5):1–7. 10.5365/wpsar.2024.15.5.1060. (**PMID: 38510816; PMCID: PMC10948340**). 38510816 10.5365/wpsar.2024.15.5.1060 PMC10948340

[44] WHO Collaboration: Partnerships. World Health Organization. https://www.who.int/about/collaboration/partnerships. Accessed 27 Jan 2025

[45] US funding cuts threaten global health response, WHO chief warns. UN News. 2025. https://news.un.org/en/story/2025/02/1160081. Accessed 21 Feb 2025

[46] WHO, Jack Ma Foundation donation for Caribbean Community countries. WHO. 2020. https://www.who.int/news-room/feature-stories/detail/who-jack-ma-foundation-donation-for-caribbean-community-countries. Accessed 21 Feb 2025

[47] Jack Ma and Alibaba Foundations Donate COVID-19 Medical Equipment to African Union Member States. AfricaCDC. WHO. 2020. https://africacdc.org/news-item/jack-ma-and-alibaba-foundations-donate-covid-19-medical-equipment-to-african-union-member-states/. Accessed 21 Feb 2025

[48] The medical humanitarian needs in Ukraine remain as urgent as ever. Médecins Sans Frontières. WHO. 2025. https://www.msf.org. Accessed 21 Feb 2025

[49] Rachmawati E, Umniyatun Y, Rosyidi M, Nurmansyah MI. The roles of Islamic Faith-Based Organizations on countermeasures against the COVID-19 pandemic in Indonesia. Heliyon. 2022;8(2):e08928. 10.1016/j.heliyon.2022.e08928. (**Epub 2022 Feb 10**). 35165662 10.1016/j.heliyon.2022.e08928 PMC8828437

[50] UNRWA. Muslim Aid.. unrwa. 2015. https://www.unrwa.org/our-partners/private-partners/partnerships/muslim-aid. Accessed 21 Feb 2025

[51] Fuster V, Frazer J, Snair M, Vedanthan R, Dzau V. Committee on Global Health and the future of the United States: a report of the national academies of sciences, engineering and medicine. The future role of the United States in global health: emphasis on cardiovascular disease. J Am Coll Cardiol. 2017;70(25):3140–56. 10.1016/j.jacc.2017.11.009. (**Epub 2017 Nov 30**). 29198877 10.1016/j.jacc.2017.11.009

[52] kFF. The U.S. President’s Emergency Plan for AIDS Relief (PEPFAR). 2024. https://www.kff.org/global-health-policy/fact-sheet/the-u-s-presidents-emergency-plan-for-aids-relief-pepfar/. Accessed 23 Feb 2025

[53] Shah, S. CIA organised fake vaccination drive to get Osama bin Laden’s family DNA. The Guardian. 2011. https://www.theguardian.com/world/2011/jul/11/cia-fake-vaccinations-osama-bin-ladens-dna. Accessed 21 Feb 2025

[54] Shah, K. CIA’s hunt for Osama bin Laden fuelled vaccine hesitancy in Pakistan. New Scientist. 2021. https://www.newscientist.com/article/2277145-cias-hunt-for-osama-bin-laden-fuelled-vaccine-hesitancy-in-pakistan/. Accessed 21 Feb 2025

[55] Snider, T. The US Has a Long History of Weaponizing Aid to Other Countries. Thruthout. 2020. https://truthout.org/articles/the-us-has-a-long-history-of-weaponizing-aid-to-other-countries/. Accessed 21 Feb 2025

[56] Report Brief. The U.S. Commitment to Global Health: Recommendations for the Public and Private Sectors. Institute of Medicine.. 2009. https://nap.nationalacademies.org/resource/12642/The%20US%20Commitment%20to%20Global%20Health%20Report%20Brief.pdf?utm_source=chatgpt.com. Accessed 21 Feb 2025

[57] Kruk ME, Gage AD, Joseph NT, Danaei G, García-Saisó S, Salomon JA. Mortality due to low-quality health systems in the universal health coverage era: a systematic analysis of amenable deaths in 137 countries. Lancet. 2018 Nov 17;392(10160):2203–2212. 10.1016/S0140-6736(18)31668-4. Epub 2018 Sep 5. Erratum in: Lancet. 2018 Nov 17;392(10160):2170. 10.1016/S0140-6736(18)32337-7. PMID: 30195398; PMCID: PMC6238021. 10.1016/S0140-6736(18)31668-4 PMC6238021 30195398

[58] Smith E, Haustein S, Mongeon P, et al. Knowledge sharing in global health research–the impact, uptake and cost of open access to scholarly literature. Health Res Policy Sys. 2017;15:73. 10.1186/s12961-017-0235-3. 10.1186/s12961-017-0235-3 PMC5576373 28851401

[59] Institute of Medicine (US) Committee on the US Commitment to Global Health. The US Commitment to Global Health: Recommendations for the Public and Private Sectors. Washington (DC): National Academies Press (US); 2009. 1, Introduction. https://www.ncbi.nlm.nih.gov/books/NBK23794/20662131

[60] World Health Organization. WHO comments on United States’ announcement of intent to withdraw. Who.int. World Health Organization: WHO; 2025. https://www.who.int/news/item/21-01-2025-who-comments-on-united-states--announcement-of-intent-to-withdraw

[61] Jha, S., Carter III, R. Global Health Security Lessons from the U.S. Biodefense Response. Think Global Health. 2024. https://www.thinkglobalhealth.org/article/global-health-security-lessons-us-biodefense-response#:~:text=In%20the%20future%2C%20the%20U.S.,military%20and%20civilian%20health%20systems. Accessed 24 Feb 2025

[62] World Health Organization. The United States of America and the World Health Organization: Partners in Global Health. www.who.int.2022.https://www.who.int/about/funding/contributors/usa

[63] WPAB. Prioritizing Global Health: World Health Organization’s Role–fundsforNGOs. Funds–Grants and Resources for Sustainability. 2024. https://www2.fundsforngos.org/donor-agencies/prioritizing-global-health-world-health-organizations-role/. Accessed 26 Jan 2025

[64] Jha, AK. Defunding WHO: Why The President’s Decision Makes America Less Safe. Health Affairs. 2020. https://www.healthaffairs.org/content/forefront/defunding-why-president-s-decision-makes-america-less-safe. Accessed 24 Feb 2025

[65] Hu, S., Denchak, M. Paris Climate Agreement: Everything You Need to Know. 2025 Jan. 23. NRDC. https://www.nrdc.org/stories/paris-climate-agreement-everything-you-need-know. Accessed 24 Feb 2025

[66] Nauert H. The United States Withdraws From UNESCO. Press Statement, U.S, Department of State. 2017. https://2017-2021.state.gov/the-united-states-withdraws-from-unesco/#:~:text=On%20October%2012%2C%202017%2C%20the,permanent%20observer%20mission%20to%20UNESCO. Accessed 24 Feb 2025

[67] Rotary International donates $14 million to sustain Polio Eradication. WHO Nigeria. https://www.afro.who.int/countries/nigeria/news/rotary-international-donates-14-million-sustain-polio-eradication-efforts-nigeria. Accessed 27 Jan 2025

[68] African countries commit to ending all forms of polio at regional meeting. United Nations. https://www.un.org/africarenewal/news/african-countries-commit-ending-all-forms-polio-regional-meeting. Accessed 27 Feb 2025

[69] Africa Kicks Out Wild Polio. WHO Africa–Polio: Global Eradication Initiative. https://www.africakicksoutwildpolio.com/. Accessed 27 Feb 2025

[70] U.S. President’s Malaria Initiative Announces Plans to Expand to New Partner Countries. U.S. President’s Malaria Initiative (PMI). 2023. https://www.pmi.gov/u-s-presidents-malaria-initiative-announces-plans-to-expand-to-new-partner-countries/. Accessed 27 Jan 2025

[71] PMI at USAID. A child has a better chance of survival now than at any other point in history–in large part, because of progress to end malaria. USAID. https://www.usaid.gov/global-health/health-areas/malaria/pmi. Accessed 27 Jan 2025

[72] COVID-19– Sub-Saharan Africa | Fact Sheet #5, Fiscalyear 2022. USAID. https://www.usaid.gov/sites/default/files/2022-09/COVID_Regional_Fact_Sheet_5_-_SSA.pdf. Accessed 27 Jan 2025

[73] Gu D, Andreev K, Dupre ME. Major trends in population growth around the world. China CDC Wkly. 2021;3(28):604–13. 10.46234/ccdcw2021.160. (**PMID:34594946 PMCID:PMC8393076**). 34594946 10.46234/ccdcw2021.160 PMC8393076

[74] Migration and migrants: a global overview. IOM UN migration. 2025. https://worldmigrationreport.iom.int/what-we-do/world-migration-report-2024-chapter-2/international-migrants-numbers-and-trends. Accessed 23 Feb 2025

[75] Stein RA. Political will and international collaborative frameworks in infectious diseases. Int J Clin Pract. 2015;69(1):41–8. 25585896 10.1111/ijcp.12489 PMC7165922

[76] World Health Organization. The United States of American and the World Health Organization: Partners in Global Health. www.who.int.2022.https://www.who.int/about/funding/contributors/usa

[77] Reality check team. Coronavirus: what are president trump’s charges against the WHO? 2020 BBC News. https://www.bbc.com/news/world-us-canada-52294623. Accessed 23 Feb 2025

[78] John TJ, Vashishtha VM. Eradicating poliomyelitis: India’s journey from hyperendemic to polio-free status. Indian J Med Res. 2013;137(5):881–94 (**PMID: 23760372; PMCID: PMC3734678**). 23760372 PMC3734678

[79] Public health milestones through the years. World Health Organization. https://www.who.int/campaigns/75-years-of-improving-public-health/milestones#year-1945

[80] Gostin LO, Meier BM, Pace L. A world Without WHO—a crossroads for US global health leadership. JAMA. 2025. 10.1001/jama.2024.28827. 39823211 10.1001/jama.2024.28827

[81] kFF. The U.S. Government and Global Health. 2024. https://www.kff.org/global-health-policy/fact-sheet/the-u-s-government-and-global-health/. Accessed 23 Feb 2025

[82] Wolf J, Johnston RB, Ambelu A, Arnold BF, Bain R, Brauer M, Brown J, Caruso BA, Clasen T, Colford JM Jr, Mills JE, Evans B, Freeman MC, Gordon B, Kang G, Lanata CF, Medlicott KO, Prüss-Ustün A, Troeger C, Boisson S, Cumming O. Burden of disease attributable to unsafe drinking water, sanitation, and hygiene in domestic settings: a global analysis for selected adverse health outcomes. Lancet. 2023;401(10393):2060–71. 10.1016/S0140-6736(23)00458-0. (**Epub 2023 Jun 5**). 37290458 10.1016/S0140-6736(23)00458-0 PMC10290941

[83] Oguntola S. WHO: Tuberculosis resurges as top infectious disease killer in 2023. Nigerian Tribune. 2024. https://tribuneonlineng.com/who-tuberculosis-resurges-as-top-infectious-disease-killer-in-2023/. Accessed 24 Feb 2025

[84] Sulaiman SK, Musa MS, Tsiga-Ahmed FI, Dayyab FM, Sulaiman AK, Bako AT. A systematic review and meta-analysis of the prevalence of caregiver acceptance of malaria vaccine for under-five children in low-income and middle-income countries (LMICs). PLoS ONE. 2022;17(12): e0278224. 10.1371/journal.pone.0278224. 36455209 10.1371/journal.pone.0278224 PMC9715017

[85] Rolle ML, Kanmounye US, Corley J, Park KB, McClain CD. Letter: the withdrawal of the United States from the World Health Organization and its impact on global neurosurgery. Neurosurgery. 2020;88(3):E281–2. 10.1093/neuros/nyaa496. 10.1093/neuros/nyaa496 33370816

[86] Humble W. World Health Organization: A Setback for Global Health or a Blessing in Disguise?. Arizona Public Health Association. 2025. WHO. https://azpha.org/2025/01/23/u-s-withdrawal-from-the-world-health-organization-a-setback-for-global-health-or-a-blessing-in-disguise/. Accessed 24 Feb 2025

[87] Huang Y. U.S. WHO Exit Could Expand China’s Influence. Think Global Heath. 2025. https://www.thinkglobalhealth.org/article/us-who-exit-could-expand-chinas-influence. Accessed 24 Feb 2025

[88] Closser S, Neel AH, Gerber S, Alonge O. From legacy to integration in the Global Polio Eradication Initiative: looking back to look forward. BMJ Glob Health. 2024;9(5): e014758. 10.1136/bmjgh-2023-014758. (**PMID:38770815;PMCID:PMC11085807**). 38770815 10.1136/bmjgh-2023-014758 PMC11085807

[89] Gandhi AR, Bekker LG, Paltiel AD, Hyle EP, Ciaranello AL, Pillay Y, Freedberg KA, Neilan AM. Potential clinical and economic impacts of cutbacks in the president’s emergency plan for AIDS relief program in South Africa: a modeling analysis. Ann Intern Med. 2025. 10.7326/ANNALS-24-01104. 39932732 10.7326/ANNALS-24-01104 PMC11996594

[90] Freedman AM, Kuester SA, Jernigan J. Evaluating public health resources: What happens when funding disappears? Prev Chronic Dis. 2013;8:10. 10.5888/pcd10.130130. 10.5888/pcd10.130130 PMC3830924 24229573

[91] National Association of County and City Health Officials. Impact of budget cuts on local health department HIV, STI, and viral hepatitis programs. One-Pager Sentinel Network Query. Washington, DC: NACCHO; April 2018. https://www.naccho.org/programs/community-health/infectious-disease/hiv-sti/hiv-sti-and-viral-hepatitis-sentinel-network

[92] Action for Global Health. Briefing: Impact of Official Development Assistance Cuts to Global Health. 2021. https://actionforglobalhealth.org.uk/wp-content/uploads/2021/06/AFGH-Briefing_Impact-of-Official-Development-Assistance-Cuts-to-Global-Health.pdf. Accessed 18 Feb 2025

[93] Smith C. How have cuts to overseas aid affected the control of malaria and other diseases. The UK Parliament. House of Lords Library. 2022. https://lordslibrary.parliament.uk/how-have-cuts-to-overseas-aid-affected-the-control-of-malaria-and-other-diseases/https://lordslibrary.parliament.uk/how-have-cuts-to-overseas-aid-affected-the-control-of-malaria-and-other-diseases/. Accessed 18 Feb 2025

[94] McDade KK, Mao W, Prizzon A, Huang RW, Ogbuoji O. United Kingdom aid cuts: implications for financing health systems. Front Public Health. 2023;10(11):1096224. 10.3389/fpubh.2023.1096224. (**PMID:37234765;PMCID:PMC10205994**). 10.3389/fpubh.2023.1096224 PMC10205994 37234765

[95] Massuda A, Hone T, Leles FAG, de Castro MC, Atun R. The Brazilian health system at crossroads: progress, crisis and resilience. BMJ Glob Health. 2018;3(4):e000829. 10.1136/bmjgh-2018-000829. (**PMID: 29997906; PMCID: PMC6035510**). 29997906 10.1136/bmjgh-2018-000829 PMC6035510

[96] eHealth Network. US Withdrawal from WHO: Implications for Global Health and India’s Healthcare Industry–Elets eHealth. eHealth Magazine. 2025. https://ehealth.eletsonline.com/2025/01/us-withdrawal-from-who-implications-for-global-health-and-indias-healthcare-industry

[97] WHO. Working groups. World Health Organization. https://www.who.int/initiatives/gap-f/working-groups#:~:text=Provides%20technical%20support%20as%20needed,contribute%20to%20national%20pharmacovigilance%20efforts.

[98] Stoltzfus H. Reflecting on the US Withdrawal from the World Health Organization. Infection Control Today. 2025. https://www.infectioncontroltoday.com/view/reflecting-on-the-us-withdrawal-from-the-world-health-organization

[99] Tennant JP, Waldner F, Jacques DC, Masuzzo P, Collister LB, Hartgerink CH. The academic, economic and societal impacts of open access: an evidence-based review. F1000Res. 2016;5:632. 10.12688/f1000research.8460.3. 27158456 10.12688/f1000research.8460.1 PMC4837983

[100] Ruger JP, Yach D. The global role of the World Health Organization. Glob Health Gov. 2009;2(2):1–11 (**PMID: 24729827; PMCID: PMC3981564**). 24729827 PMC3981564

[101] Tamasiga P, Onyeaka H, Umenweke GC, Uwishema O. An extended SEIRDV compartmental model: case studies of the spread of COVID-19 and vaccination in Tunisia and South Africa. Annals Med Surger. 2023;85(6):2721–30. 10.1097/MS9.0000000000000627. 10.1097/MS9.0000000000000627 PMC10289697 37363551

[102] Raviglione M, Maher D. Ending infectious diseases in the era of the Sustainable Development Goals. Porto Biomed J. 2017;2(5):140–2. 10.1016/j.pbj.2017.08.001. (**Epub 2017 Aug 30**). 32258607 10.1016/j.pbj.2017.08.001 PMC6806806

[103] WHO. The NTD road map: together towards 2030. World Health Organization. 2025. https://www.who.int/teams/control-of-neglected-tropical-diseases/overview

[104] WHO. The End TB strategy. World Health Organization. 2016. https://www.who.int/teams/global-tuberculosis-programme/the-end-tb-strategy#:~:text=The%20WHO%20Global%20Tuberculosis%20Programme,care%2C%20multisectoral%20action%20and%20innovation.

[105] WHO Collaborating Centre for Tuberculosis. Reducing the burden, leading to a target of ending TB by 2035. The University of Sydney. https://www.sydney.edu.au/medicine-health/our-research/research-centres/who-collaborating-centre-on-tuberculosis.html#:~:text=The%20newly%20designated%20World%20Health,TB%20(DR%2DTB)

[106] Aborode AT, David KB, Uwishema O, Nathaniel AL, Imisioluwa JO, Onigbinde SB, Farooq F. Fighting COVID-19 at the expense of malaria in Africa: the consequences and policy options. Am J Trop Med Hyg. 2021;104(1):26–9. 10.4269/ajtmh.20-1181. 33205743 10.4269/ajtmh.20-1181 PMC7790111

[107] Pradhan AU, Uwishema O, Wellington J, Nisingizwe P, Thambi VD, Onyeaka CVP, Onyeaka H. Challenges of addressing neglected tropical diseases amidst the COVID-19 pandemic in Africa: a case of chagas disease. Annals Med Surger. 2022;81:104414. 10.1016/j.amsu.2022.104414. 10.1016/j.amsu.2022.104414 PMC9392556 36035600

[108] Shehu NY, Okwor T, Dooga J, Wele AM, Cihambanya L, Okonkon II, et al. Train-the-trainers intervention for national capacity building in infection prevention and control for COVID-19 in Nigeria. Heliyon. 2023;9(11): e21978. 10.1016/j.heliyon.2023.e21978. 38034678 10.1016/j.heliyon.2023.e21978 PMC10682610

[109] WHO. Training for emergencies. World Health Organization. 2023. https://www.who.int/emergencies/training

[110] WHO. Capacity building. World Health Organization. 2025. https://www.who.int/teams/digital-health-and-innovation/capacity-building

[111] Board of Health Science Policy, Institute of Medicine, National Academies of Sciences, Engineering, and Medicine. Washington (DC). Health systems strengthening: Building Day-to-Day care and public health capacities. Global Health Risk Framework–NCBI Bookshelf. 2016. https://www.ncbi.nlm.nih.gov/books/NBK367953/

[112] DeCorby-Watson K, Mensah G, Bergeron K, Abdi S, Rempel B, Manson H. Effectiveness of capacity building interventions relevant to public health practice: a systematic review. BMC Public Health. 2018. 10.1186/s12889-018-5591-6. 29859075 10.1186/s12889-018-5591-6 PMC5984748

[113] World Health Organization (WHO). EvalCommunity Jobs & Experts. CEBRI Revista. 2024. https://www.evalcommunity.com/donors-and-nonprofit/world-health-organization-who/. Accessed 17 Feb 2025.

[114] Youde J. Philanthropy and Global Health. McInnes C, Lee K, Youde J, editors. The Oxford Handbook of Global Health Politics. 2018; 408–25. 10.1093/oxfordhb/9780190456818.013.24. Accessed 27 Jan 2025

[115] Shiffman J. Donor funding priorities for communicable disease control in the developing world. Health Policy Plann. 2006;21(6):411–20. 10.1093/heapol/czl028. 10.1093/heapol/czl028 16984894

[116] Reubi D. Epidemiological accountability: philanthropists, global health and the audit of saving lives. Econ Soc. 2018;47(1):83–110. 29805316 10.1080/03085147.2018.1433359 PMC5950534

[117] Matee MI, Manyando C, Ndumbe PM, et al. European and developing countries clinical trials partnership (EDCTP): the path towards a true partnership. BMC Public Health. 2009;9:249. 10.1186/1471-2458-9-249. 19619283 10.1186/1471-2458-9-249 PMC2719636

[118] Fernández Ó, Kissack R. The European union’s role in global health: embracing governance complexity? The European Union in International Affairs. 2024;13:117–45.

[119] Jones CM, Sobngwi-Tambekou J, Mijumbi RM, Hedquist A, Wenham C, Parkhurst J. The roles of regional organisations in strengthening health research systems in Africa: activities, gaps, and future perspectives. Int J Health Policy Manage. 2022;11:2672. 10.34172/ijhpm.2022.6426 PMC9818106 35279037

[120] Kumaresan J, Huikuri S. Strengthening Regional Cooperation, Coordination, and Response to Health Concerns in the ASEAN Region: Status, Challenges, and Ways Forward. 2015. https://www.eria.org/ERIA-DP-2015-60.pdf

[121] Sands P. Putting country ownership into practice: the global fund and country coordinating mechanisms. Health Syst Reform. 2019;5(2):100–3. 31194640 10.1080/23288604.2019.1589831

[122] Abdul S, Adeghe EP, Adegoke BO, Adegoke AA, Udedeh EH. Public-private partnerships in health sector innovation: Lessons from around the world. Magna Scientia Adv Bio Pharm. 2024;12(1):045–59.

[123] Yang T, Xu B. Incentive effects of government subsidy on technological innovation: Evidence from pharmaceutical industry. Finance Res Lett. 2023;55:103928.

[124] Uwishema O, Nchasi G, Goodluck Nnko G, Mtawala E, Boniphace Bulimbe D, Haidari Kassim G, Mushi J, Nazir A, Angeline Peñamante C. The insight through the current immunotherapeutic guidelines for infectious diseases. Int J Surger. 2023;109(1):71–2. 10.1097/JS9.0000000000000152. 10.1097/JS9.0000000000000152 PMC10389434 36799800

[125] Borger J. US gives G7 countries a list of reforms it wants WHO to undertake. The Guardian. 2020. https://www.theguardian.com/world/2020/apr/30/us-gives-g7-countries-a-list-of-reforms-it-wants-who-to-undertake. Accessed 14 Feb 2025.

[126] Rakhra K. The G20 as a multilateral force. CEBRI revista. 2024. https://cebri.org/revista/br/artigo/133/the-g20-as-a-multilateral-force. Accessed 25 Jan 2025.

[127] World Health Organization. Joint statement of the United States of America and the World Health Organization on the U.S.-WHO strategic dialogue. www.who.int.2022.https://www.who.int/news/item/27-09-2022-joint-statement-of-the-united-states-of-america-and-the-world-health-organization-on-the-u.s.-who-strategic-dialogue

[128] Zhao AP, Li S, Cao Z, Hu PJH, Wang J, Xiang Y, et al. AI for science: predicting infectious diseases. J Safety Sci Resil. 2024;5(2):130–46.

[129] Ekundayo F. Using machine learning to predict disease outbreaks and enhance public health surveillance. World J Adv Res Rev. 2024;24(3):794–811.

[130] Wikipedia contributors. Satmed. Wikipedia, The Free Encyclopedia; [cited 2025 Feb 14]. https://en.m.wikipedia.org/wiki/Satmed. Accessed 14 Feb 2025.

[131] McVeigh K. South Sudan medics trial AI software to identify snake and improve bite treatment. The Guardian; 2024. https://www.theguardian.com/global-development/2024/sep/25/south-sudan-medics-trial-ai-software-to-identify-snake-and-improve-bite-treatment. Accessed 14 Feb 2025.

[132] Einaste T. Blockchain and healthcare: the Estonian experience. e-Estonia. 2018. https://e-estonia.com/blockchain-healthcare-estonian-experience/. Accessed 19 Feb 2025.

[133] Tagde P, Tagde S, Bhattacharya T, Tagde P, Chopra H, Akter R, Kaushik D, Rahman MH. Blockchain and artificial intelligence technology in e-Health. Environ Sci Pollut Res Int. 2021;28(38):52810–31. 10.1007/s11356-021-16223-0. (**Epub 2021 Sep 2**). 34476701 10.1007/s11356-021-16223-0 PMC8412875

[134] Chung IB, Caldas C. Applicability of blockchain-based implementation for risk management in healthcare projects. Blockchain in Healthcare Today. 2022. 10.30953/bhty.v5.191 PMC9907422 36779025

[135] Mieras LF, Taal AT, Post EB, Ndeve AGZ, Van Hees CLM. The development of a mobile application to support peripheral health workers to diagnose and treat people with skin diseases in resource-poor settings. Tropic Med Infect Dis. 2018;3(3):102. 10.3390/tropicalmed3030102 PMC6160956 30274498

[136] Suffescom Solutions. Blockchain healthcare management cost. Suffescom solutions. https://www.suffescom.com/blog/blockchain-healthcare-management-cost

[137] Mahmoud K, Jaramillo C, Barteit S. Telemedicine in low- and middle-income countries during the COVID-19 pandemic: a scoping review. Front Public Health. 2022;22(10): 914423. 10.3389/fpubh.2022.914423. (**PMID:35812479;PMCID:PMC9257012**). 10.3389/fpubh.2022.914423 PMC9257012 35812479

[138] Ajekiigbe VO, Ogieuhi IJ, Odeniyi TA, et al. Understanding Nigeria’s antibiotic resistance crisis among neonates and its future implications. Discov Public Health. 2025;22:28. 10.1186/s12982-025-00422-y.

[139] George NS, David SC, Nabiryo M, Sunday BA, Olanrewaju OF, Yangaza Y, Shomuyiwa DO. Addressing neglected tropical diseases in Africa: a health equity perspective. Glob Health Res Policy. 2023;8(1):30. 10.1186/s41256-023-00314-1. (**PMID:37491338;PMCID:PMC10367333**). 37491338 10.1186/s41256-023-00314-1 PMC10367333

[140] Yaffe H. Cuban medical internationalism: a paradigm for South-South cooperation. Int J Cuban Stud. 2023;15(2):203–34. 10.13169/intejcubastud.15.2.0203.

[141] White F. Primary health care and public health: foundations of universal health systems. Med Princ Pract. 2015;24(2):103–16. 10.1159/000370197. (**Epub 2015 Jan 9**). 25591411 10.1159/000370197 PMC5588212

[142] Oleribe OO, Momoh J, Uzochukwu BS, Mbofana F, Adebiyi A, Barbera T, Williams R, Taylor-Robinson SD. Identifying key challenges facing healthcare systems in Africa and potential solutions. Int J Gen Med. 2019;6(12):395–403. 10.2147/IJGM.S223882. (**PMID:31819592;PMCID:PMC6844097**). 10.2147/IJGM.S223882 PMC6844097 31819592

[143] Langlois EV, McKenzie A, Schneider H, Mecaskey JW. Measures to strengthen primary health-care systems in low- and middle-income countries. Bull World Health Organ. 2020;98(11):781–91. 10.2471/BLT.20.252742. (**Epub 2020 Sep 28**). 33177775 10.2471/BLT.20.252742 PMC7607465

[144] Worsley-Tonks KE, Bender JB, Deem SL, Ferguson AW, Fèvre EM, Martins DJ, et al. Strengthening global health security by improving disease surveillance in remote rural areas of low-income and middle-income countries. Lancet Glob Health. 2022;10(4):e579–84. 35303467 10.1016/S2214-109X(22)00031-6 PMC8923676

[145] Bollyky TJ, Templin T, Cohen M, Dieleman JL. Lower-income countries that face the most rapid shift in noncommunicable disease burden are also the least prepared. Health Aff. 2017;36(11):1866–75. 10.1377/hlthaff.2017.0708. 10.1377/hlthaff.2017.0708 PMC7705176 29137514

[146] Hanson K, Brikci N, Erlangga D, Alebachew A, De Allegri M, Balabanova D, et al. The lancet global health commission on financing primary health care: putting people at the centre. Lancet Glob Health. 2022;10(5):e715–72. 35390342 10.1016/S2214-109X(22)00005-5 PMC9005653

[147] Liu Y, Hall BJ, Ren M. How the US presidential election impacts global health: governance, funding, and beyond. Glob Health Res Policy. 2024;9:49. 10.1186/s41256-024-00391-w. 39563373 10.1186/s41256-024-00391-w PMC11577883

[148] Thomas KK. The other time a U.S. president withheld WHO funds: johns hopkins Bloomberg school of public health. 2020. https://publichealth.jhu.edu/2020/the-other-time-a-us-president-withheld-who-funds.

[149] U.S. News & World Report. Who funds the World Health Organization? A list of its biggest donors. 2025. https://www.usnews.com/news/health-news/articles/2025-01-28/who-funds-the-world-health-organization-a-list-of-its-biggest-donors. Accessed 12 Feb 2025.

[150] Jack Ma Foundation and Alibaba Foundation donate to seven more countries in Asia to fight COVID-19. Business Wire; 2020. https://www.businesswire.com/news/home/20200328005024/en/Jack-Ma-Foundation-and-Alibaba-Foundation-Donate-to-Seven-More-Countries-in-Asia-to-Fight-COVID-19. Accessed 14 Feb 2025.

[151] Wikipedia contributors. Serum Institute of India. Wikipedia, The Free Encyclopedia. Wikimedia Foundation. https://en.m.wikipedia.org/wiki/Serum_Institute_of_India. Accessed 14 Feb 2025.

[152] Taylor LA, Aveling EL, Roberts J, Bhuiya N, Edmondson A, Singer S. Building resilient partnerships: how businesses and nonprofits create the capacity for responsiveness. Front Health Serv. 2023;15(3):1155941. 10.3389/frhs.2023.1155941. (**PMID:37256212;PMCID:PMC10225548**). 10.3389/frhs.2023.1155941 PMC10225548 37256212

[153] Maani N, Van Schalkwyk MC, Petticrew M, Ralston R, Collin J. The new WHO foundation–global health deserves better. BMJ Glob Health. 2021;6(2): e004950. 10.1136/bmjgh-2021-004950. (**PMID:33547178;PMCID:PMC7871260**). 33547178 10.1136/bmjgh-2021-004950 PMC7871260

